# UpStart Parent Survey: A New Psychometrically Valid Tool for the Evaluation of Prevention-focused Parenting Programs

**DOI:** 10.1007/s10995-012-1152-2

**Published:** 2012-10-11

**Authors:** Karen Benzies, Dawn Clarke, Leslie Barker, Richelle Mychasiuk

**Affiliations:** 1 Faculty of Nursing, University of Calgary, Calgary, AB Canada; 2 Department of Child and Youth Studies, Mount Royal University, Calgary, AB Canada; 3 Provincial Early Childhood Team, Alberta Health Services, Calgary, AB Canada

**Keywords:** Measurement, Child development, Intervention, Parenting

## Abstract

Parents are the most significant influence on the growth and development of young children. All parents can increase their knowledge of developmental milestones and parenting practices by participating in effective programs that offer information and support. However, there is limited outcome evaluation of programs offering these services. Prevention-focused parenting programs (P-FPPs) are key frontline services designed to educate parents and improve the overall well-being of children. Evaluation of these programs is currently weak; this is not to say they are ineffective, rather that their effectiveness has been poorly evaluated. Rigorous evaluation of P-FPPs would support informed funding and evidence-based policy decisions. The purpose of this study was to conduct a preliminary psychometric analysis of the *UpStart Parent Survey* (USPS)—a tool developed specifically for evaluating this type of program. Preliminary analysis revealed uni-dimensionality of each scale, strong internal consistency and temporal stability, as well as strong concurrent validity on 9 of the 11 items examined with an urban Canadian population. In its first round of psychometric evaluation, the USPS demonstrated promise as a brief, easy to administer, scientifically rigorous tool for the evaluation of prevention-focused parenting programs.

## Introduction

Over the past decade, there has been a dramatic and essential demand for accountability with evidence-based programming. Currently, most parenting programs have limited evidence of their effectiveness [1, 2], with the majority of research literature limited to intervention programs targeted at high risk children and families (e.g., [3, 4]). Prevention-focused parenting programs (P-FPPs) are key front-line services that help parents learn about child development and healthy parent/child relationships, introduce valuable support networks, and promote parental connection to their community. In order to thrive, P-FPPs must demonstrate they achieve their intended outcomes and improve parental functioning.

The foundational capacities for lifelong learning and health are established early in life [5]. Parents have the potential to significantly contribute to the healthy development of young children [5] and most parents will benefit from additional information, support, or guidance. Research indicates that there is a substantial gap in what parents *believe* they know and what they *need* to know to support their child’s development [6]. P-FPPs are designed to serve as primary and secondary prevention supports, aimed at educating, increasing resiliency, improving relationships between parents and their children, and promoting parental and family competence, in populations who are not in crisis. The goal of most P-FPPs is to build parenting capacity to prevent problems before they occur, by increasing protective factors such as knowledge of child development, healthy parenting skills, and parental competence and satisfaction [7]. While community-based P-FPPs are well-positioned to address these needs and support growing families, are they actually realizing these objectives?

Rigorous evaluation requires the use of reliable and valid measurement tools. In a community setting, program providers and program funders often have conflicting goals and opposing ideology when defining effective programs and useful measurement tools. While both are committed to collecting meaningful data, program providers prefer evaluations that are unobtrusive, quickly completed, and easy to interpret. Conversely, program evaluators favor measurement tools that have strong psychometric properties and present an accurate assessment of program outcomes. Typically, one group’s goal is met at the expense of the other, as scientifically rigorous measures tend to be long and burdensome [8]. Furthermore, instruments generally examine a single outcome variable, such as self-efficacy [9] or parenting stress [10] and avoid the evaluation of broad concepts. The ideal tool for community based programs would be a brief measurement instrument that evaluated multiple outcome variables, while maintaining low respondent burden and strong psychometric properties.

Decades of research have shown that specific factors enhance positive parent/child relationships and increase the likelihood of positive child outcomes [11]. In response to this research, a consensus conference was held in Canada and a set of outcomes common to effective parenting programs were identified. The consensus group used prior literature relevant to their regional context to recommend specific outcomes important for effective parenting education programs [12]. The common outcomes generated were: quality of life, self-efficacy, family functioning, social support, parenting knowledge and skills, parental competence, emotional health, parenting stress, and formal and informal support systems. These specific indicators were used in a Western Canadian urban setting as the foundation for the UpStart Parent Survey (USPS).

The purpose of this study was to conduct a preliminary psychometric analysis of the *USPS.* The USPS contained three separate but inter-related subscales: Parenting Knowledge, Parenting Experiences, and Program Satisfaction. Constructs measured by the USPS include; parenting knowledge and skills, self-efficacy, mental health, social support, parenting stress, and family functioning, These concepts are strongly linked to successful parenting and child well-being, and are expected to be improved in effective P-FPPs. The psychometric evaluation investigated (a) internal consistency reliability of the USPS, (b) temporal stability (test–retest) reliability, and (c) concurrent validity of individual items on the USPS *Parenting Experiences* subscale with established measurements previously deemed psychometrically valid and reliable.

## Methods

### Participants

Participants (345 parents/caregivers of young children) were recruited at P-FPPs between April 2010 and March 2011. Program leaders were oriented to the project by the research team prior to April 2010. Parent participants who completed only the *USPS* filled out the survey at the end of the last P-FPPs session and returned the USPS to the program leader. The program leader then returned all of the surveys to the research office in the stamped, addressed envelope provided.

Once the process was well-established, program facilitators randomly recruited a subset of the participants to complete the second and third component of the study—a test/retest sample (n = 22), and a ‘gold-standard’ concurrent validity sample (n = 53). The ‘gold-standard’ component of the study included all of the validation measures; participants completed the USPS and the validation measures for psychometric analysis. For each of the components, consenting parents were given instructions for each measure along with an addressed, stamped envelope to return their packages to the research office. Parent participants who completed component two or three were mailed a $20CDN gift card to acknowledge their time. The study was approved by the Mount Royal University, Human Research Ethics Board and the University of Calgary, Conjoint Health Research Ethics Board.

As there was no way to account for the demographic characteristics of non-responders to determine if they were eligible to participate or not, we were unable to accurately calculate a response rate [13]. Socio-demographic characteristics of participants are reported in Table 1. The subset of participants randomly selected to complete the additional two elements used for the concurrent validity subset of this experiment exhibited small but statistically significant differences from those who filled out the Upstart Parent Survey alone. Parents/caregivers who completed the additional components were on average younger (*p* = .03), made less money (*p* = .05), and were less likely to use English as a first language at home (*p* = .01). Of the total participant population, approximately 72 % were married, 71 % were Caucasian, and more than half reported a household income greater than $80,000.

**Table 1:** Characteristics of individuals participating in the prevention-focused education programs who completed an USPS

Characteristic	n*	Frequency	Percentage (%)
*Age*	345		
Under 18		1	0.3
18–29 years		85	24.6
30–39 years		205	59.4
40–49 years		47	13.6
50 years or over		7	2.0
*Gender*	342		
Female		308	90.1
*Marital status*	344		
Single		30	8.7
Common law		59	17.2
Divorced/separated		12	3.5
Married		241	70.1
Widowed		2	0.6
*Level of education*	344		
Less than high school		40	11.6
High school diploma		30	8.7
Certificate/diploma after high school		46	13.3
College/university degree		228	66.1
*Household income*	314		
Less than $20,000/year		52	16.6
$20,000–$40,000/year		33	10.5
$40,000–$80,000/year		64	20.4
More than $80,000/year		165	52.5
*English as a first language*	343		
Yes		289	84.3
*Ethnicity*	224		
Aboriginal		19	5.9
Arab/West Asian		3	0.9
Black		6	1.7
Chinese		20	6.2
Filipino		24	7.0
Japanese		4	1.2
Korean		1	0.3
Latin American		6	1.9
South Asian		5	1.5
South East Asian		3	0.9
Caucasian		231	71.3
Other		2	0.6
*Birth parent of child*	345		
Yes		322	93.3
*Number of children/household*	296		
None		17	5.7
One		136	45.9
Two		118	39.9
Three or more		25	8.5

* *n’*s vary due to missing data

### Prevention-focused Parenting Programs

Six different agencies offering 10 separate programs for parents of young children participated in this study. The parenting programs contributing to this investigation were diverse, and most had previously demonstrated evidence of effectiveness on outcomes, such as parenting morale, social support, and parenting roles and responsibilities. All programs underwent program evaluation processes that included reliably measurable outcomes. Programs targeted parents of children 6 years of age and under, and were located in geographically diverse areas of the city. The programs were offered for a defined length of time that varied from 4 to 11 weeks; weekly classes lasted between 2 and 3 h.

The primary focus of all programs was education and support, and each program had well-established curricula tailored to their parenting population. Typical child growth and development, as well as parenting strategies for young children, were essential elements to each program. Programs varied their dissemination style; some programs included only a classroom/parent discussion and learning component, others include both classroom and parent/child learning components.

#### Target Measure

##### UpStart Parent Survey

The UpStart Parent Survey (USPS) was designed as a brief, paper and pencil, self-report measure of common outcomes expected of P-FPPs. The USPS is comprised of three subscales: (1) Parenting Knowledge/Skills (PK), (2) Parenting Experience (PE), and (3) Program Satisfaction (PS). The survey takes approximately 15 min to complete.

The Parenting Knowledge/Skills subscale (PK) is a 10 item scale that captures concepts such as growth and development, discipline strategies, child health and safety, and parental responses to everyday challenges. These items were uniquely developed by the research team from the P-FPP curricula. The PK scale uses a 7-point Likert scale ranging from 1 (*Strongly Disagree*) to 7 (*Strongly Agree*). Scores on individual items were summed to create a total scale score. The theoretical range of scores is 10–70 with higher scores indicating greater parenting knowledge and skills. Each item on the PK scale offers a *Not Covered* response option for concepts that were not addressed in the specific program curriculum. *Not Covered* responses were analyzed as missing values.

Using a similar 7-point Likert scale, [1 (*Strongly Disagree*) to 7 (*Strongly Agree*)], the Parenting Experiences subscale (PE) includes 11 items that capture additional common outcomes of parenting programs including parenting self-efficacy, emotional health, social support, parenting stress, and family functioning. The PE items were designed by the researchers from the common outcomes identified at the consensus conference, and were subsequently validated in this study with standardized measures. Scores on individual items were summed to create a PE total scale score. The theoretical range of scores is 11–77 with higher scores indicating a more positive parenting experience.

The PK and PE subscales were designed as a post-test/retrospective pre-test. The parent reports a “today” score and a “before this program” score for each item that assesses both post-test and retrospective pre-test particulars. To clarify, a retrospective pre-test is administered at the same time as the post-test but asks respondents to reflect upon their level of understanding or skill prior to the intervention/training. A post-test/retrospective pre-test [14] survey design is recommended when the goal of program evaluation is (a) the assessment of individual perceptions of change [15], (b) establishment of trust [16], conservation of limited program time, and (c) provider guided reflection on personal growth related to the program [17]. Post-test/retrospective pre-tests provide parents with an opportunity to reflect on how much they have learned over the course of the program.

The Program Satisfaction (PS) subscale contains 7 items that capture engagement in, and satisfaction with the program. The PS utilizes a 5-point Likert scale ranging from, *strongly disagree* to *strongly agree*. Scores on individual items were summed to create a PS total score. The theoretical range of scores is 7–35 with higher scores indicating greater satisfaction with the parenting program.

#### Validation Measures

Validation measures were selected by the consensus committee to align with aspects of the PE scale, as well as exhibit (a) strong psychometric properties, (b) suitability to a wide ranging demographic, and (c) ability to capture constructs critical to social support, parenting efficacy, and family functioning [12].

##### Brief Family Assessment Measure: General Scale

The brief FAM-General Scale [18] is a short version (14-item) of the FAM scale that provides an overall rating of family functioning. The scale can be administered and scored in under 10 min; responses range from 0 (*strongly agree*) to 3 (*strongly disagree*). Items are summed and scores translated to T-scores. Higher scores indicate disrupted family functioning. Test–retest reliability is .56–.66 over 12 days, and internal consistency was reported at .86–.94 [18]. Cronbach’s alpha for this study was found to be .73.

##### Parenting Morale Index

The Parenting Morale Index (PMI) [19] is a 10-item, self-report, paper and pencil measure designed to capture psychological energy, positive parenting spirits, and enthusiasm for parenting. Six items are reverse scored, but all items range from 1 (*not at all*) to 5 (*very often*). Items are summed to create a total score. Higher scores indicate higher parenting morale. Cronbach’s alpha has been reported at .86 [19], and was .82 for this study.

##### Family Support Scale

The Family Support Scale (FSS) is a self-report measure that assesses the helpfulness of formal and informal social support sources for parents raising young children [20]. The scale includes 20 items designed to measure social support through examination of informal kinship, social organization items, formal kinship, immediate family, specialized professional services, and generic professional services. Respondents rate social support on a 5-point Likert scale ranging from 1 (*not helpful*) to 5 (e*xtremely helpful*). The FSS was scored by summing items: higher scores indicate greater social support [20]. Cronbach’s alpha has been previously reported as .85 [21]. For this study, Cronbach’s alpha was .76.

##### *SF*-*8 Health Survey*

The SF-8 health survey is a shortened version of the SF-36, and is designed to assess 8 domains of health, each with a single item: physical functioning, role limitations due to physical health, bodily pain, general health perceptions, vitality, social functioning, role limitations due to emotional problems, and mental health [22]. Each survey produces a psychometrically-based physical component summary and a mental component summary. The SF-8 is scored and normalized so 50 is the average score or norm. Scores well below average are problematic and indicate poor health [23]. Cronbach’s alphas for this study were .66 (physical component summary), .80 (mental component summary), and .83 (total survey).

Tool to Measure Parenting Self-Efficacy *(TOPSE Parenting Evaluation).* The TOPSE is a self-report, paper and pencil measure designed to assess parents’ perceived ability to manage their children [9]. The tool was originally created to evaluate education programs for parents. The TOPSE has 8 separate subsections comprised of 48 items, of which 6 are reverse scored. Respondents rate items using a 10-point Likert scale ranging from 0 (*completely disagree*) to 10 (*completely agree*). Items are summed to create a total score; higher scores indicate greater parenting self-efficacy. Cronbach’s alphas ranged from .69 to .85 for each of the subscales [9]. Cronbach’s alphas for this study ranged from .62 to .91.

### Data Analysis

Data were examined for errors, outliers, and patterns of missing values. For the *USPS* missing values (up to 2 missing value per subscale) were replaced with the mean score for the individual participant on the specific subscale. This, however, was rarely employed, as there were few missing responses (0.6–5.7 %) on any of the subscales. Data were not markedly skewed for any measure. Significance was set at *p* < .05 for all statistical tests. All analyses were conducted using SPSS 19.0 software for Windows. Descriptive statistics for all measures were calculated. Using principal components method without rotation, we conducted factor analysis using Bartlett’s Test of Sphericity and scree plots on the items for each subscale of the USPS. Internal consistency reliability was assessed using Cronbach’s alpha and an alpha greater than .70 is deemed acceptable for a new scale [24]. Pearson’s correlations were used to assess convergent validity between the target and validation measures [24]. Cohen’s [25] guidelines were used to interpret the strength of the correlations (i.e., small = .10, medium = .30, and large = .50).

## Results

Descriptive statistics, reliabilities, and percentile scores for the USPS are presented in Table 2. Inter item correlation analysis revealed correlations between the items ranging from .11–.55, .14–.70, and .58–.88 on the PK, PE, and PS subscales respectively. For each scale, Bartlett’s Test of Sphericity was significant. Visual examination of the scree plots identified a sharp discontinuity after one for all subscales suggesting a single component. One principal component with an eigenvalue equal to 1.0 or greater suggests uni-dimensionality of a scale. The percent of variance explained by the principal components were as follows: 44.47 for the PK scale, 48.04 for the PE scale, and 74.25 for the PS scale. For the PK scale the proportion of variance explained by one of the items (*I make time to read with my child everyday*) was very low (.16), which suggests that this item was not well represented on this scale. Similarly for the PE scale, one item (*I know who to call and where to go in the community when I need help*) was low (.08). A large standard deviation for scores on these items suggests they may need to be reworded for the next iteration of the USPS.

**Table 2:** Descriptive statistics, reliabilities, and percentiles, for the Upstart Survey subscales

Upstart Survey Subscales	Number of items	Full sample^a^			Percentile^a^			Test–retest^b^
		α	*M*	*SD*	80th	90th	98th	
Parenting knowledge	10	.87	53.35	8.47	60	63	67	.72
Parenting experience	11	.91	56.19	11.28	66	70	75	.76
Satisfaction	7	.94	32.71	4.04	35	35	35	.87

^a^n = 345

^b^n = 22

Internal consistencies were strong exceeding criterion established prior to analysis. In addition, temporal stability for the USPS scales was satisfactory. Correlations between scores on the USPS and the validation measures are presented in Table 3. Based on Cohen’s [23] guidelines for interpreting strength of correlations, there was a large correlation, in the expected direction, for the USPS Parenting Experience Item: *My emotional health is good*—and the *SF*-*8 Mental Scale*. Additionally, there were large to medium correlations, in the expected directions, for eight of the PE scale items and their validation measures. Only two PE items failed to demonstrate significant correlation with the intended validation measure. The PE items may not capture the same constructs as the validation measures, resulting in small correlation. The Brief FAM failed to show any correlation with USPS items suggesting it may not be an ideal candidate for concurrent validity testing of items on the PE subscale or that these items may need to be slightly modified.

**Table 3:** Correlations between caregiver scores on parenting experience target items and baseline validation measures

Parenting experience items	Pearson’s *r* correlation	Outcome measure
I have confidence in my parenting skills	.42**	TOPSE: Self acceptance
I feel positive in my role as a parent	.40**	Parenting Morale Index
I know who to call and where I can go in the community when I need help	.34*	TOPSE: Learning and knowledge
I know where I can get answers to my parenting questions	.36**	TOPSE: Learning and knowledge
I have someone to talk to when I need support	.44**	Parenting Morale Index
I am able to manage stress	.47**	SF-8: Mental Component Score
I know ways to meet my family’s needs with the money and resources I have	.44**	TOPSE: Control Subscale
My emotional health is good (that is, I do not feel anxious, depressed or irritated)	.60**	SF-8: Mental Component Score
I know how to “speak up” for what my family and children need	.11	FAM Brief
I feel supported by my partner in my parenting (if you parent alone, please check “not covered”)	.34*	Family Support Scale
In our family, we take the time to listen to each other	−.24	FAM Brief

* *p* < .05; ** *p* < .01. *n* = 53

## Discussion

This study contributes to community-based research by presenting and evaluating a simple, efficient, program evaluation tool. The *Upstart Parent Survey* is a new measure of common outcomes expected to be achieved following participation in an effective parenting education program. In its first round of psychometric evaluation, the USPS demonstrated promise as a brief, easy to administer measure for prevention-focused parenting programs (P-FPP). The uni-dimensionality of the survey’s PK, PE, and PS subscales reinforced our hypothesis that each scale measured a single concept. The internal consistency reliabilities for each scale were strong for a new measure. Temporal stability over 2 weeks for the post-test scale scores was satisfactory.

Concurrent validity for most items was moderate to strong. Two variables, PK: *I make time to read with my child everyday* and PE: *I know who to call and where to go in the community when I need help* exhibited low shared variance. Further investigation of these two items illustrated that the PK item was worded inappropriately; following additional investigation the authors believe that “everyday” induced defensive responding by the participants. The PE item was double barreled; the item asks 2 separate questions in one statement. In order to address these weaknesses, these constructs will be corrected when an updated version of the USPS is drafted. There were very few missing values on the PK, PE, or PS subscales of the USPS. This suggested that parents found the USPS acceptable for providing information about themselves and their families. This also indicated that language and reading level of the survey were accessible to a culturally diverse population.

The current study is limited by parental perceptions of change related to the program. Parental perceptions of change may be biased. Future studies should include observational measures of parent–child interaction, or health and other social indictors such as validated cases of child maltreatment. The study was also limited by sample size of some of the components, and a sample that was primarily mothers. Future studies should increase the number of fathers sampled because parental responses between fathers and mothers may vary based on differences in paternal expectations.

Future versions of the USPS may wish to reverse score some of the items to prevent convenience response bias based upon high scores for all items being in the same column. This allows researchers to distinguish whether or not participants are appropriately reading each question. Although the majority of the sample population was Caucasian, there was enough ethnic diversity to permit initial generalization to multi-cultural regions. This, however, will require further investigation. Subsequent evaluation of the USPS will also include a traditional pre-test/post-test group that will be appropriately compared to a retrospective pre-test group. In addition, cognitive interviews with a small number of respondents will be carried out to ensure participants and evaluators are referring to comparable concepts.

Owing to time constraints and respondent burden, retrospective pre-test/post-test designs are a pragmatically desirable approach to capturing program evaluation of P-FPPs. Although proponents of the traditional pre-test challenge the ‘accuracy’ of the retrospective pre-test, evidence supports the validity of this type of assessment under certain circumstances [26]. The results from this study indicate that the USPS may be a sufficient tool to evaluate effectiveness of the ability of P-FPPs to improve parenting knowledge and skills, and key outcomes associated with improved family functioning. As funding becomes increasingly dependent upon demonstration of effective programming, the need for rigorous but easy to adminster evaluation tools has become apparent. Despite its minor limitations, the *UpStart Parent Survey* offers prevention-focused parenting programs an efficient, psychometrically valid measurement tool that can be implemented in its current form.
